# Tricuspid valve myxoma in a patient with congestive heart failure

**DOI:** 10.1186/1757-1626-3-21

**Published:** 2010-01-12

**Authors:** Enrico Vizzardi, Antonio D'Aloia, Ermannna Chiari, Matilde Nardi, Gregoriana Zanini, Roberto Cabras, Giacomo Faden, Cristian Maiandi, Livio Dei Cas

**Affiliations:** 1 Section of Cardiovascular Disease. Department of Applied Experimental Medicine, University Study of Brescia, Brescia, Italy

## Abstract

Myxomas are the most frequent benign primary cardiac tumours (50% of benign heart tumours). This kind of tumour is most likely to be localized in the left atrium, followed by the right atrium, right ventricle and left ventricle. Quite exceptional is the presence of a myxoma originating from the tricuspid valve or from the Eustachian valve. We describe the case of a woman with moderate dyspnoea of unknown origin and the presence of tricuspid myxoma who underwent tricuspid valve curettage.

## Background

Myxomas are the most frequent benign primary cardiac tumours (50% of benign heart tumours). They are most commonly detected in people between 30 and 60 years of age, although published findings report congenital myxomas, tumours developed during childhood and even in extremely old patients. This kind of tumour is most likely to be localized in the left atrium (75%), followed by the right atrium (18%), right ventricle (4%) and left ventricle (3%) [1,2]. Quite exceptional is the presence of a myxoma originating from the tricuspid valve [3,4] or from the Eustachian valve [5].

## Case Report

**Figure 1:**
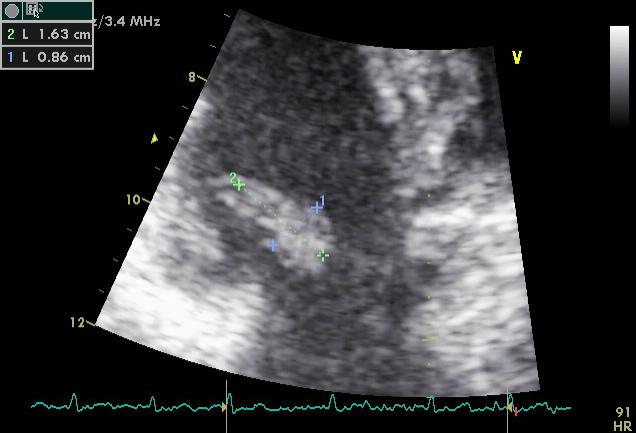
**Echocardiographic image of tricuspid mass**.

**Figure 2:**
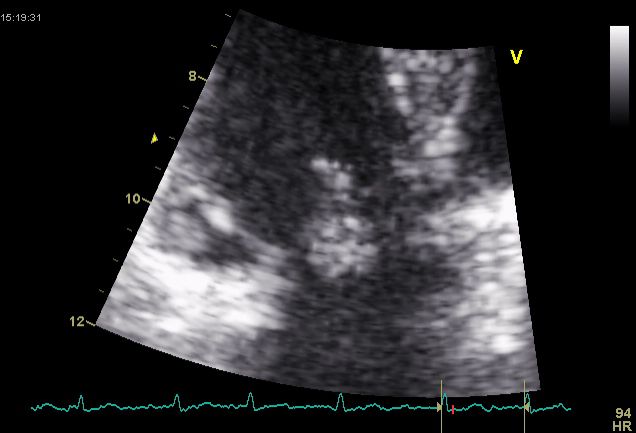
**Image of mass through the tricuspid valve**.

A 44-year-old woman was evaluated for moderate dyspnoea of unknown origin. She had already undergone other clinical and instrumental tests that had ruled out pulmonary genesis. Transthoracic echocardiography revealed a mobile hyperlucent mass measuring 0.9 × 1.6 cm, with suspected tumoral myxoma located on the septal leaflet of the tricuspid valve and moving through and from the tricuspidal annulus (Figs. 1 and 2), with moderate tricuspid regurgitation and mild valve stenosis (mean gradient 8 mmHg, systolic pulmonary artery 45 mmHg). The patient underwent chest X-ray examination and thoracic CT, revealing no pathological condition.

Surgical removal with tricuspid valve curettage was performed to improve the symptoms of congestive heart failure and prevent pulmonary embolism. Histological examination of the mass confirmed the diagnosis of myxoma and its benign nature. The postoperative course was uneventful and follow-up examination showed complete normalization of the Doppler echocardiographic parameters and the absence of clinical symptoms (i.e. dyspnoea).

## Discussion

Myxomas are rarely localized in the right heart originating from the right atrium or from the tricuspid valve; they usually present symptoms related to low cardiac output or to pulmonary arterial hypertension with hepatomegaly, oedema, ascites and cyanosis. This clinical picture varies, however, with the position of the tumour. The literature reports patients who were symptomatic for syncopes and dyspnoea [1,2] or embolic manifestations that usually involved the lungs [6,7]. In the case of friable polypoid myxomas there may also be multiple pulmonary embolism [8], frequently mistaken for a septic embolus, which can lead to severe pulmonary hypertension and sometimes also to aneurisms of the pulmonary arteries [9]. Lastly, in the case of a right-to-left shunt at an atrial level, paradoxical embolism of the brain and kidneys and aortic bifurcation may occur [10]. Resection of the tumour constitutes the definitive treatment for cardiac myxomas, using different techniques based on the location of the tumour in order to control embolization. In the case of tricuspid valve myxomas, tumour excision, tricuspid valvuloplasty or valve replacement may also be necessary. Surgery mortality is around 1% and relapses are possible (1-5% of all cases). Recurrences are usually due to metastatic dissemination or incomplete resection of the lesion and arise within 48 months of the operation [11-13]. Our clinical case highlights once more the absence and the non-specificity of the clinical manifestation of cardiac myxomas, especially when located in the right heart. Furthermore, as well established in the literature, this case stresses the importance and reliability of two-dimensional echocardiograpy for diagnosis of the tumour mass thanks to direct visualization of the tricuspid leaflets and normalization of the haemodynamic parameters.

## Consent

Written informed consent was obtained from the patient for publication of this case report and accompanying images. A copy of the written consent is available for review by The Editor-in-Chief of the journal

## Competing interests

The authors declare that they have no competing interests.

## Authors' contributions

EV RC GF CM participated in case design and coordination, AD EC MN LD performed echocardiography and clinical evaluation of the patient. All author read and approved the final manuscript
